# All Quiet on the Far Western Front

**Published:** 1990-09

**Authors:** Martin Crosfil


### ALL QUIET ON THE FAR WESTERN FRONT

This correspondent, having been released on parole after 37 years in the N.H.S. is reduced to enquiring from erstwhile colleagues as to what has happened on the health scene in Cornwall in the last few months. So far the answers have been identical "Nothing".

"Nothing" includes the formal opening of the new operating theatre in Penzance with its immediate closure for relaying of the floor. One of the few useful things a central authority could do (but I'll bet it doesn't) would be to keep a record of the number of times this particular problem has arisen; it is by no means unique.

"Nothing" of course also includes some Sisyphean scurrying on the administrative side in preparation for the latest Great Leap Foreweard (1). Close followers of the Cornish scene will know that originally there were to be three self governing trusts covering mental health, the acute services and what's-left ("community"). Somewhere between Truro and Bristol the acute services scheme was subjected to Solomonization (2) but as this threatened non-viability (and as one of the hospitals concerned but not consulted was not even in the N.H.S.) the "timetable has been revised" and this leaves the community trust way out ahead of the field. The Poo Bah of this trust is to be Surgeon Vice Admiral Sir Godfrey Milton Thomson. Rumour has it that it was necessary to choose somebody who could outrank the General Manager, a mere unfrocked Wing Commander but, mirabile dictu, he is also medically qualified which should in theory make no difference but could prove a very present help in time of trouble.

Under the stirring slogan "A terminal in every toilet" the emplacement of the technological infrastructure proceeds apace (some of the jargon sticks). The consultant body has, of course been persuaded that this is a Good Thing as it will facilitate clinical audit. My personal reservation is based on a semantic twist. I believe that "audit" to an administrator means cost analysis whereas a doctor hopes to assess the quality of patient care. I suspect that funding will cease once the first of these is achieved. Incidentally, on the subject of semantics, one has got used to avoiding the use of such phrases as "serous exudate" in front of a patient for fear of being misunderstood. Similarly the nurses will have attendants or assistants (or handmaidens?) rather than a ward full of aides. Had we not better be careful with the number of terminals we have around?

(1) Plot on log paper the dates 1858 1911 1948 1967 1974 1982 1989 and predict the date of the next reorganisation.
(2) 1 Kings 3 25

MARTIN CROSFIL

